# Protocol to calculate aircraft emissions for international air routes in South America

**DOI:** 10.1016/j.xpro.2022.101952

**Published:** 2022-12-16

**Authors:** Qiang Cui, Yilin Lei, Ye Li

**Affiliations:** 1 School of Economics and Management, Southeast University, Nanjing 211189, China; 2 School of Business Administration, Nanjing University of Finance and Economics, Nanjing 210023, China

**Keywords:** Energy, Chemistry, Earth sciences

## Abstract

Recently, aviation pollution has drawn important social attention. The protocol proposed in this paper can simultaneously calculate the overall emissions of six aviation pollutants (CO_2_, CO, HC, NOx, SO_2_, and PM2.5), including the landing and take-off emissions and climb/cruise/descent emissions. The international routes in South America during 2019–2021 are an example to illustrate the use of this protocol. This protocol can provide a methodological basis for calculating aviation pollutant emissions in different countries and regions.

For complete details on the use and execution of this protocol, please refer to Cui et al. (2022b).^1^

## Highlights

• Unify the CO_2_ and non-CO_2_ calculations using the modified BFFM2-FOA-FPM technique
• Calculate overall emissions for international routes in South America
• Calculate aviation pollutant emissions in different countries and regions

## Abstract (editor highlights)

Publisher’s note: Undertaking any experimental protocol requires adherence to local institutional guidelines for laboratory safety and ethics.

## Graphical abstract

**Figure:**
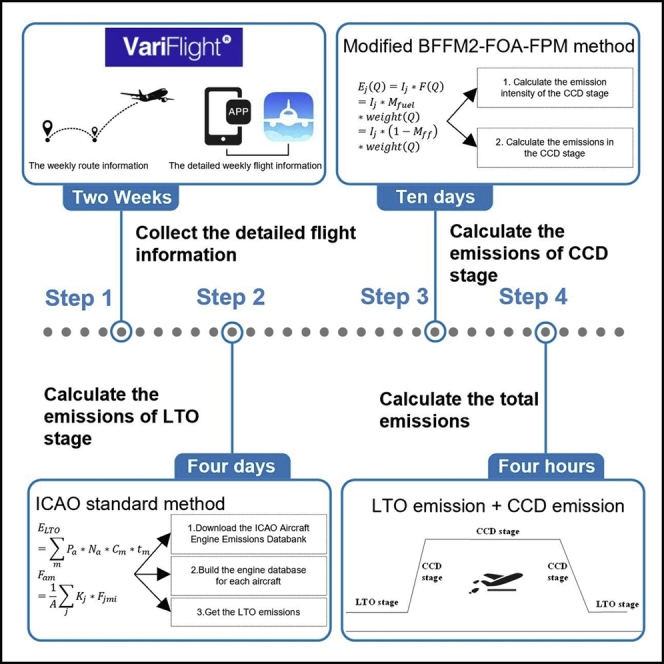

## Before you begin

### Method scope

The rapid growth of air travel has led to the intensification of climate impacts. The main factors that affect climate change, such as contrails, fuel consumption, aviation emissions, etc., have attracted more and more attention.^2^ In addition to rapid growth, aviation emissions have other characteristics. First, the aircraft cruising stage is generally more than 10,000 meters, which is too far from the ground vegetation and cannot be transformed easily. Second, the retention time is long, and the harm of mixing with particulate matter is more than twice the harm of carbon dioxide emitted. Third, although aircraft generally fly in the stratosphere, there is still much water vapor, suspended solid particles, impurities, etc., which are easy to mix with carbon dioxide, increasing the harm.^3^^,^^4^ Generally, the flight process consists of seven steps: Engine Starting, Taxiing, Taking Off, Climbing, Cruising, Descending, and Landing.^5^ It is usually divided into the Landing and Take-Off (LTO) cycle and the Climb/Cruise/Descent (CCD) stage. Therefore, the overall emissions include LTO emissions and CCD emissions. Thus, the protocol is a method for simultaneous accounting of six major aviation pollutants (CO_2_, CO, HC, NOx, SO_2_, and PM2.5) emissions. The technique applies to CO_2_ contaminants and non-CO_2_ pollutants from aviation kerosene combustion. The focus of the protocol is to provide a systematic accounting method for assessing the overall aviation pollutant emissions, including CCD emissions and LTO emissions. It can provide guidance for aviation environment researchers to collect and calculate aviation emissions and provide basic data and methods for policy research of aviation environment.

### Method structure

**Figure 1:**
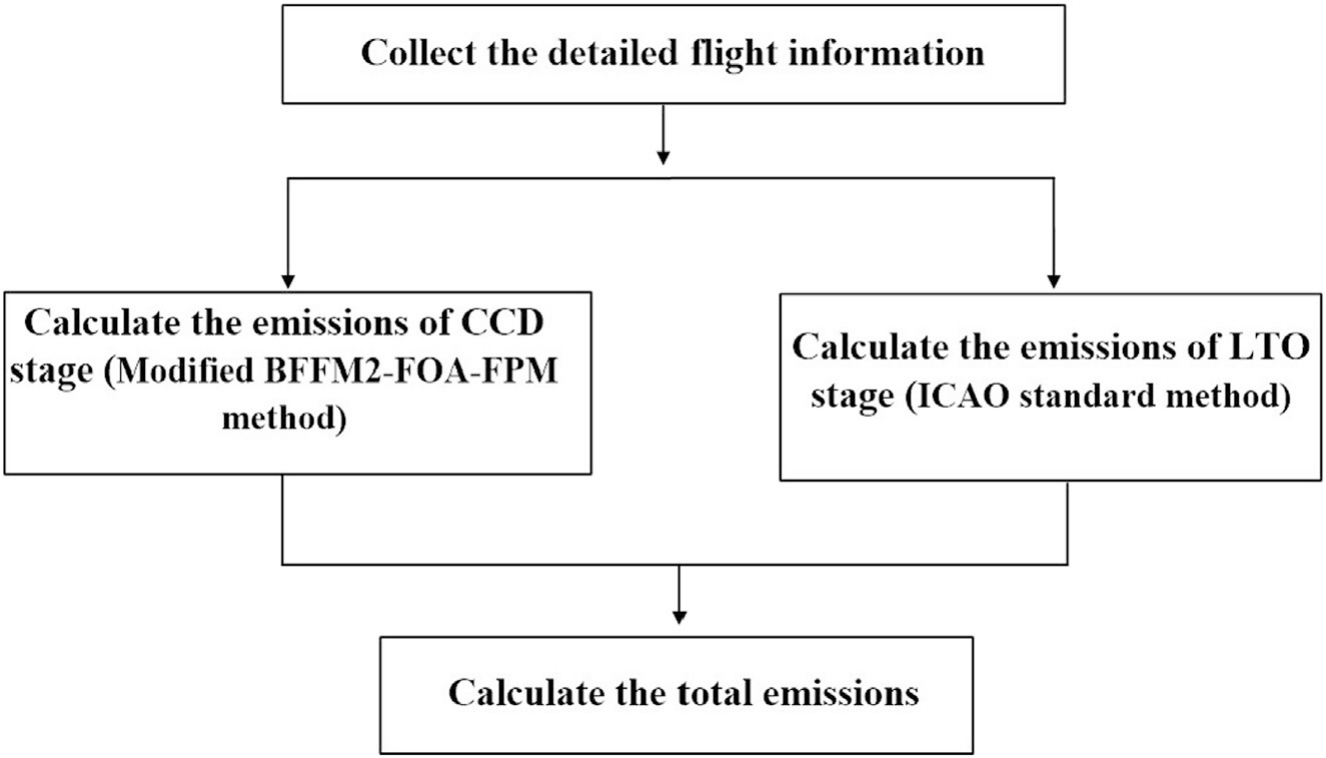
Detailed steps of the method

Because the current methods of accounting for the emissions of aviation pollutants primarily focus on the accounting of CO_2_ and lack detailed large-scale application examples,^1^ it is challenging to control the emission of aviation pollutants in any region. Therefore, we propose a modified method to calculate the overall aircraft emissions. The CCD emissions are calculated through the Modified BFFM2-FOA-FPM method, and the LTO emissions are calculated based on the ICAO Aircraft Engine Emissions Databank.^6^ Figure 1 summarizes the methods proposed in this paper.

### Comparison with other methods

Aiming at the accounting method of gaseous pollutant emission of aero-engine during standard landing and take-off cycle, ICAO has successfully developed simple, advanced, and complex methods according to different calculation methods and data requirements since the 1970s. For example, based on the ICAO calculation system, the U.S. Environmental Protection Agency (EPA) puts forward the EPA (Environmental Protection Agency) method combined with the actual situation. Meanwhile, the European Environment Agency (EEA) has established the EMEP (European monitoring and evaluation program) cooperative action framework. Since then, ICAO has further improved the calculation method and proposed the ICAO carbon emission calculator, which can estimate the aviation emission per unit passenger based on the data of various aircraft types.

1. ICAO emission inventory method.

ICAO emission inventory calculation methods are divided into simple, advanced, and complex methods, and simple methods are divided into A and B.^7^ Simple method A takes the number of LTO cycles and the emission index of various emissions as an essential reference, which can quickly evaluate the emissions during the aircraft LTO cycle. Still, the model does not consider the aircraft type’s impact and each stage’s operation time. Simple method B further considers the variation characteristics of fuel flow rate and emission index in different operation stages and uses the operation time set by ICAO under other rated thrusts in different settings. The emission index was found from the EEDB (Engine Emissions Data Bank) database to calculate each aircraft’s emission of various pollutants. Researchers have carried out many studies on the establishment of aviation emission inventory and pollution simulation based on simple methods: Kesgin^8^ and Altuntas^9^ approximate estimated the aircraft emissions from domestic routes of multiple airports in Turkey and determined the emission characteristics of air pollutants in different operation stages of aircraft. Cokorilo^10^ estimated aircraft engines' emission and carbon emission costs at Belgrade airport, Serbia, and proposed corresponding emission reduction plans.

In the advanced method, considering that the fuel flow rate and emission index will change with external thrust setting, flight level, atmospheric environment, and other factors in actual operation, the BFFM2 (Boeing Fuel Flow Method II) method is proposed for correction. Turgut et al.^11^ used ICAO advanced method to quantify the emission of gaseous pollutants from thirty airports in Turkey.

The complex method is to apply the measured accurate data, performance information, operation time, fuel consumption, and thrust of an aero-engine under different loads and external conditions to the complex computer model to obtain the final pollutant emission. For example, Xu et al.^12^ estimated the engine pollutant emissions of Shanghai Hongqiao International Airport and Shanghai Pudong International Airport in the taxiing phase based on the Aircraft Communication Addressing and Reporting System (ACARS) data.

The direct use of model reference value in ICAO’s simple methods will uncertainty the accounting results. On the other hand, the advanced and complex methods further improved have highly accurate results. However, they have the limitations of high data requirements, complex implementation, high research cost, and are unsuitable for mass calculation. Therefore, the development of relevant research is relatively slow.

2. American EPA method.

American EPA method mainly aims to calculate emission inventory during the LTO cycle.^13^ Its overall calculation idea is the same as ICAO’s simple method B. Still, the difference is that the EPA method considers the correction of the maximum mixing layer height on the operation time of the aircraft during climbing and approach under different meteorological conditions. The air pollutant emission above the maximum mixing layer height will not affect the air quality near the ground. The ultimate mixing layer height is taken as the effective emission height of the aircraft, and the relationship between meteorological conditions and aero-engine emission is established. Zhou et al.^14^ estimated the pollutant emission during the take-off and landing cycle of domestic civil aviation aircraft in 2015 based on the improved EPA method. They compared the calculation results with the calculation results of the guide method, providing a technical reference for revising the calculation method of aircraft emission in the guide. And Baxter et al.^15^ used this method to calculate the air pollutant emissions of aircraft engines at Kansai International Airport in Japan.

Although the EPA method considers meteorological conditions and establishes the relationship between meteorological conditions and aero-engine emissions, it is helpful to understand the relationship between meteorological conditions and emissions. However, the calculation of emission inventory during the aircraft LTO cycle by the EPA method is the same as that of the ICAO simple B method. Therefore, there may be no significant difference between the two calculation results for a single aircraft. As a result, and because many organizations and projects apply ICAO recommended method, ICAO recommended method is the most effective method for LTO cycle pollutant emission evaluation.^16^

3. European EMEP method.

EMEP calculation method is divided into the level I, level II, and level III methods. Level III is divided into method A and method B.^17^ For fuel consumption calculation in the LTO cycle and cruise phase, the level I method considers the factors, including emission index and fuel consumption in-flight phase and range type. The level II method further considers the influence of aircraft type based on the level I method. Level III method A is to model the emission list, average value of fuel consumption, and flight distance during a cruise in the LTO stage and analyze the fuel consumption level of each segment, which can be used in the transportation planning of airlines. Level III method B calculates the pollutant emissions in each stage of the whole trajectory based on the aircraft’s flight performance and the engine’s rated dynamic parameters. Based on this, Pereira et al.^18^ assessed the environmental impact of replacing existing aviation fuels with hydrogen or natural gas.

This method is an accounting method of pollutant emission of aircraft during the whole flight based on fuel statistical data. Still, it focuses on analyzing the emission characteristics of aero-engine from the fuel perspective and ignores the differences between engine types.

4. The ICAO Carbon Emission Calculator.

The ICAO Carbon Emission Calculator^6^ is a universal method for calculating aviation carbon emissions. The ICAO methodology employs a distance-based approach to estimate an individual’s aviation emissions using available data on aircraft types. The ICAO Carbon Emission Calculator requires users to enter the origin and destination airport of the flight. It is then compared with the published scheduled flights to obtain the type of aircraft used to serve the two related airports and the number of departures per aircraft. Then map each plane to one of the equivalent aircraft types to calculate the trip’s fuel consumption based on the great circle distance between the airports involved in the journey. The load factor and passenger-to-freight ratio obtained from the traffic and operating data collected by ICAO are then applied to obtain the total fuel usage attributable to the passengers. Then, the system weights the average fuel consumption of the journey based on the take-off frequency of each equivalent aircraft type. The fuel consumption is then divided by the total number of economy class equivalent passengers, giving an average fuel burn per economy class passenger. The result is then multiplied by 3.157 to obtain the amount of CO_2_, which is the carbon footprint of each passenger. Some studies have focused on estimating aviation carbon emissions. For example, Wasiuk et al.^19^ estimated the global emissions during 2005–2011, but they have not involved particular aircraft, routes, and airlines. Liu et al.^20^ applied the ICAO method to estimate the emissions of the routes among 208 Chinese airports, but no discussion has been done on specific aircraft and distance segments.

However, there are some drawbacks to the approach provided by ICAO. First, the distance difference is not enough. For example, according to the VariFlight,^21^ A320-214 flew between 360 km and 3,649 km on domestic routes in China in 2018, exceeding the methodology provided by the ICAO. Second, there is no distinction between specific aircraft. ICAO’s calculation method only considers large sequences and does not consider differences between subsequences. For example, the A320 family has many families, such as the A320–100 and A320-200, with different engine types, which may lead to a significant difference in the carbon emissions of the two aircraft.^22^^,^^23^ Third, various pollutants cannot be calculated at the same time.

To sum up, the accounting methods of aviation pollutants mainly used in the existing research are primarily formulated by European and American countries. In emission accounting, most pollutant emission indexes use ICAO reference values, and the calculation model is rarely improved and modified according to the actual situation. And it is not possible to calculate different pollutants at the same time.

Generally, the primary calculation methods for non-CO_2_ emissions are the ICAO method (Boeing Fuel Flow Method 2 (BFFM2) method and First Order Approximation (FOA) method.^19^^,^^24^ Therefore, this protocol proposes a Modified BFFM2-FOA-FPM method based on the Fuel Percentage Method (FPM). This method combines the BFFM2-FOA method and the Fuel Percentage Method (FPM),^25^ which can calculate the CO_2_ and non-CO_2_ emissions simultaneously. Some papers have focused on calculating CO_2_ and non-CO_2_ emissions, but no unified approach exists to connect the two accounting methods. Furthermore, the calculation method of CO_2_ emissions has not considered the difference of sub-series, and that of non-CO_2_ emissions has not focused on the emissions in the CCD stage. Therefore, we built a new Modified BFFM2-FOA-FPM method to calculate CO_2_ and non-CO_2_ emissions from the CCD stage. The LTO emissions are calculated using the International Civil Aviation Organization (ICAO). And our approach has considered the aircraft types' emission intensity. We divide the route distance into several groups and get the six pollutions of aircraft types in these different distances to ensure that the calculation results are accurate. Compared with existing research, our results cover more detailed aircraft types, and we corrected the results with the actual flight time to make it more real. In addition, our method can calculate the PM2.5 of the CCD stage. Existing studies focus on calculating PM2.5 in the LTO stage, while there are few corresponding calculation methods for PM2.5 in the CCD stage. The Modified BFFM2-FOA-FPM method can estimate the PM2.5 value of the CCD stage.

To more clearly compare the differences and connections between the methods in this paper and other methods, the main advantages and disadvantages of each method are summarized as shown in Table 1.

**Table 1:** Main advantages and disadvantages of each method

Method	Advantage	Disadvantages
ICAO emission inventory method	1. Simple methods A can quickly evaluate the emissions during the aircraft LTO cycle; 2. Simple method B further considers the variation characteristics of fuel flow rate; 3. In the advanced method, the BFFM2 (Boeing Fuel Flow Method II) method is proposed for correction; 4. The complex method is to apply various information to the complex computer model to obtain the final pollutant emission to make the results more accurate.	1. The simple methods will uncertainty the accounting results; 2. The advanced and complex methods have the limitations of high data requirements, complex implementation, high research cost, and are unsuitable for mass calculation.
American EPA method	1. Considering meteorological conditions; 2. Establishing the relationship between meteorological conditions and aero-engine emissions.	The calculation of emission inventory during the aircraft LTO cycle by the EPA method is the same as that of the ICAO simple B method.
European EMEP method	1. This method is an accounting method of pollutant emission of aircraft during the whole flight based on fuel statistical data.; 2. Focuse on analyzing the emission characteristics of aero-engine from the fuel perspective.	Ignore the differences between engine types.
The ICAO Carbon Emission Calculator	Employ a distance-based approach to estimate an individual’s aviation emissions using available data on aircraft types.	1. The distance difference is not enough; 2. There is no distinction between specific aircraft; 3. Various pollutants cannot be calculated at the same time.
Method of this paper	1. The Modified BFFM2-FOA-FPM combines the BFFM2-FOA method and the Fuel Percentage Method (FPM), which can calculate the CO2 and non-CO2 emissions simultaneously; 2. Consider the aircraft types' emission intensity; 3. Divide the route distance into several groups to ensure that the calculation results are accurate; 4. The method can calculate the PM2.5 of the CCD stage.	1. The overall emissions are calculated through the standard LTO stage. Therefore, the emissions due to delays are not considered; 2. The aircraft transfer caused by temporary weather is not considered.

### Proposed timing

The actual time necessary to realize each calculation step will depend strongly on the timing of collecting the flight information. The more route data to be collected, the longer it takes. It should be noted that flight information can also be obtained through purchase on the Variglight, but the price is very expensive, and it is difficult to ensure that clear and detailed flight information can be obtained, which is not conducive to data processing and use. Therefore, it is not recommended to purchase directly from the official Variglight. This paper uses South America’s international routes as an example to provide the timing interval of each step.

## Key resources table

**Table:** 

REAGENT or RESOURCE	SOURCE	IDENTIFIER
**Deposited data**		

Route origin and destination data	VariFlight^21^	https://www.variflight.com/
Flight data	VariFlight^21^	https://www.variflight.com/
Information about Jet-A	EASA^26^	https://www.easa.europa.eu/domains/environment/icao-aircraft-engine-emissions-databank
Engines of each aircraft	EASA^26^	https://www.easa.europa.eu/domains/environment/icao-aircraft-engine-emissions-databank

## Step-by-step method details

### Collect the detailed flight information

**Timing: 2 weeks**

Resource levels: [Intel Core i7-8550U/16G RAM/ 64 bit Windows 10].

1. Collect the weekly route information.
  a. Log on to the website of Variglight (https://map.variflight.com/).
  b. Click “query” and enter “departure country” and “arrival country”.
  c. Start the query.
    i. Get the route information between the two countries.
    ii. Query all South American countries to get all international routes in South America, containing the origin airport and the destination airport.

***Note:*** According to our statistics, there is little change in the weekly arrangement of international routes in South America, so we collect the weekly route information. For example, when the departure country was Colombia, and the arrival country was Venezuela in 2019, we can get six routes: Medellín-Caracas, Cali-Caracas, Bogotá-Santiago Marino, Bogotá-Caracas, Medellín-Santiago Marino, and Cali-Santiago Marino.

2. Collect the detailed weekly flight information.
  a. Open the app of Variglight with the mobile phone.
  b. Query, and record all flight information by taking off and landing place, such as frequency, aircraft type, aircraft seat layout, flight time, flight distance, transit information, etc.
  c. Collect the data for all the routes. For example, the numbers of international routes in South America from 2019 to 2021 are 118, 62, and 77. This step is the most time-consuming.

***Note:*** For example, for Medellín-Caracas in 2019, we collected the following information: the flying distance is 1,049 km, the aircraft type is 737-400, the airline is Avior Airlines, and the average weekly flight frequency is 6, and the average flying time is 2 h and 3 min. Furthermore, this route has no transit flights.

### Calculate the emissions of LTO stage (ICAO standard method)

**Timing: 4 days**

Resource levels: [Intel Core i7-8550U/16G RAM/ 64 bit Windows 10].

3. Download the ICAO Aircraft Engine Emissions Databank.
  Log on to the website (https://www.easa.europa.eu/domains/environment/icao-aircraft-engine-emissions-databank), and download the latest emissions databank. The databank contains the emission data of six pollutants of all types of engines in an LTO cycle.
4. Build the engine database for each aircraft according to the aircraft type information in the flight information collected earlier. This will contain each aircraft’s engine types and engine numbers.

***Note:*** This paper uses standard aviation kerosene (Jet-A), which is also a relatively common aviation fuel. Relevant information can be found through the KRT link (Downloads-Emissions Databank (07/2021)-Gaseous Emissions and Smoke).

***Note:*** For example, for Medellín-Caracas in 2019, its aircraft type is 737-400, its engine type is CFM56-3B2, and the number is 2. Then, we can get the standard emissions of 737-400 in an LTO cycle. In a standard LTO cycle, its CO_2_ emission is 2.658194 tons, its HC emission is 674.830g, its CO emission is 11976.422g, its NOx emission is 8425.296g, its PM 2.5 emission is 170.640g, and its SO_2_ emission is 3258.54g.

5. Get the LTO emissions.

We can get the six pollutions for each aircraft in an LTO cycle based on the engine type and engine number. Then multiplying by the flight frequency and accumulating, we can get the LTO emissions.

***Note:*** For example, for Medellín-Caracas in 2019, Avior Airlines’ total LTO emissions were 1658.713 tons CO_2_, 7.473288 tons CO, 0.421094 tons HC, 5.257385 tons NOx, 0.10648 tons PM2.5, and 2.033329 tons SO_2_.

**CRITICAL:** [If one flight is a transit flight and the aircraft take off and lands twice, the LTO emissions should multiply by 2; If the aircraft takes off and lands three times, the LTO emissions should multiply by 3.]

### Calculate the emissions of CCD stage (modified BFFM2-FOA-FPM method)

**Timing: 10 days**

Resource levels: [Intel Core i7-8550U/16G RAM/ 64 bit Windows 10].

6. Calculate the emission intensity of the CCD stage.
  a. Divide the distance segments.
    The emission intensity of the same model at different distance sections is very different, so this paper first segments the distance. All routes are divided into distance segments according to distance.
    ***Note:*** For example, for the international routes in South America, we divide the distance into 11 segments based on the distance range: 0–500 km, 501 km-1,000 km, 1,001 km-1,500 km, 1,501 km-2,000 km, 2,001 km-2,500 km, 2,501 km-3,000 km, 3,001 km-3,500 km, 3,501 km-4,000 km, 4,001 km-4,500 km, 4,501 km-5,000 km, and 5,001 km–5500 km.
  b. Calculate the $ratio_{cr}$ in Modified BFFM2-FOA-FPM method for each aircraft in different distance segments.
    In the Modified BFFM2-FOA-FPM method, the primary unknown variable is $ratio_{cr}$. We comprehensively calculated it by integrating the fuel volume data per unit flying time of each aircraft type and the ICAO Aircraft Engine Emissions Databank. Then, the value is corrected using the aircraft flight time collected earlier.
  c. Get the CO_2_ emission intensity of each aircraft in each distance segment.
    If the $ratio_{cr}$ is determined, the CCD CO_2_ emissions in each route can be obtained. Based on the distance of each flight, we can get the CO_2_ emission intensity of each aircraft in each distance segment.
    **CRITICAL:** [Carbon emission intensity has two applications: 1. It is used to calculate the emissions of transit routes. 2. Used to calculate the carbon emission intensity of the other five pollutants.]
  d. Calculate the emission intensity of other five pollutants.
    Based on the CO_2_ emission intensity, we can get the fuel consumption intensity of each aircraft in each distance segment and then calculate the emission intensities of the other five pollutants.
    For SO_2_, the emission intensity is 3.870 g/kg multiplied by the fuel consumption intensity;
    For CO and HC, $I_{j}=I_{j0}\text{∗}\frac{\theta^{3.3}}{\delta^{1.02}}$. θ is the ratio of outside temperature to 288 K; δ is the ratio of external pressure to sea level pressure. $I_{j0}$ is the standard emission coefficient of an LTO stage of CO or HC (g/kg). We assume that the aircraft’s cruising altitude is 10,000 meters, then we can obtain the relationship between the altitude of 0–10,000 meters and the temperature. We use interpolation integration to get θ; According to the relationship between altitude and air pressure, the interpolation integration is used to obtain δ. According to the ICAO aircraft engine emissions databank and fuel intensity value, we obtain the emission intensity of CO and HC.
    For NOx, $I_{NOx}=I_{j0}*\frac{\delta^{0.51}}{\theta^{1.65}}\text{∗exp}\left(19.0\text{∗}\left(0.0063-\frac{0.622\text{∗}\varphi \text{∗}Pv}{P-\varphi \text{∗}Pv}\right)\right)$. $I_{j0}$ is the standard emission coefficient of a LTO stage of NOx (g/kg). θ is the ratio of outside temperature to 288 K; δ is ratio of external pressure to sea level pressure. φ is atmospheric relative humidity; P is external pressure; Pv is atmospheric saturation pressure. The value of θ, δ, *φ*, P, and Pv is also obtained through interpolation integration.
    PM2.5 can be divided into Nonvolatile Component Fine Particles (NCFP) and Volatile Component Fine Particles (VCFP), which can be calculated by Modified BFFM2-FOA-FPM.
    **CRITICAL:** [In this step, we can get a data sheet that contains the emission intensity of these six pollutants of different aircraft at different distances. This data sheet is based on the fuel emission intensity. Because the average flight distance and aircraft type are other in different regions, different regions correspond to different fuel emission intensities, which need to be recalculated for different areas and cannot be applied to all parts. It cannot be used in all regions. If we research a new region, the steps must be repeated.]

7. Calculate the emissions in the CCD stage.

The emission intensity of each aircraft type is multiplied by the flight distance, flight frequency, and other data to obtain the emission at the CCD stage.

**CRITICAL:** [If one flight is a transit flight and the aircraft take off and lands twice, the CCD emissions are the sum of two flight distances before and after; If the flight distance is three segments, the situation is the same.]

It should be noted that the calculation of CCD emission intensity needs to coordinate the situation of all routes, so it is impossible to give an example for only one route. The detailed results can be found in Tables S4, S5, and S6.

### Calculate the total emissions

**Timing: 4 h**

Resource levels: [Intel Core i7-8550U/16G RAM/ 64 bit Windows 10].

The LTO emission and the CCD emission are added to obtain the total emission.

## Expected outcomes

### Statistical characteristics of the routes

**Figure 2:**
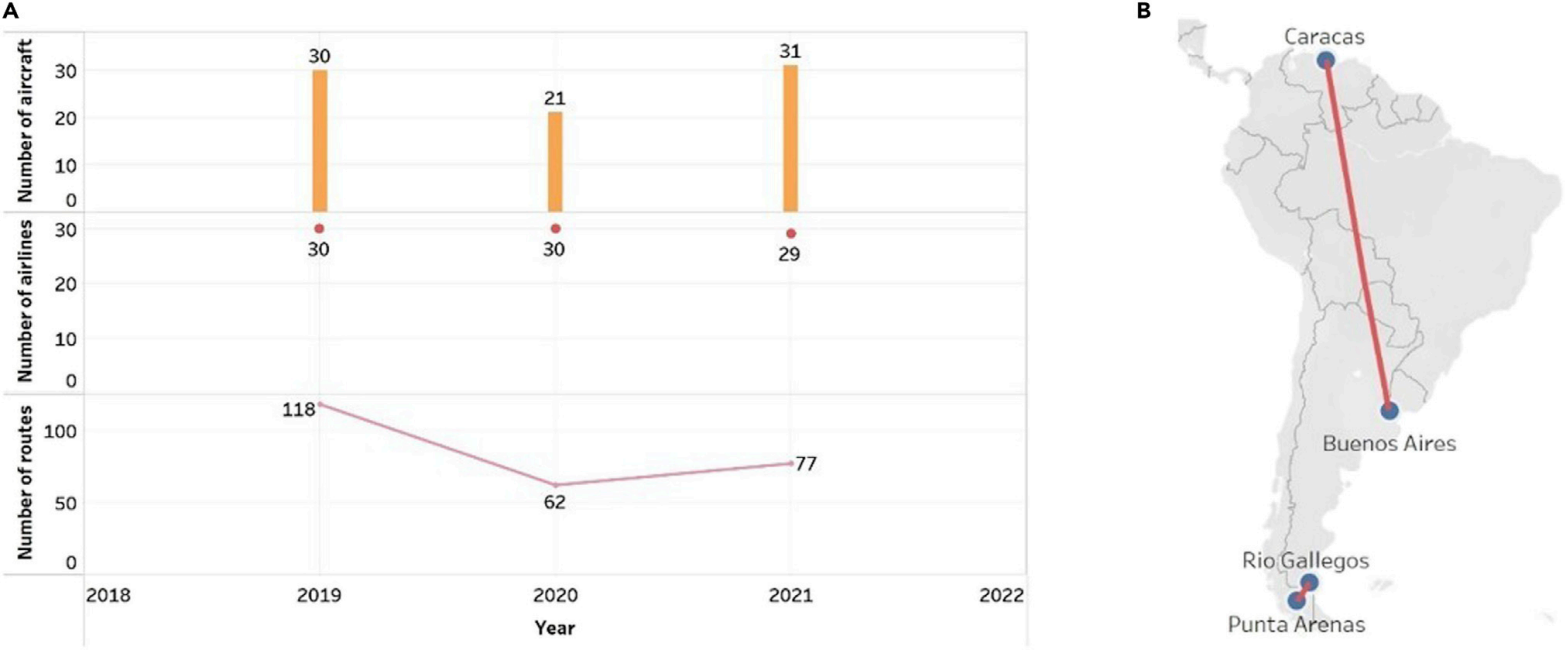
Statistical characteristics of the air routes (A) Number of airlines, number of aircraft, and number of routes. (B) The farthest route and the nearest route.

We take the information on all the international routes in South America from 2019 to 2021 as an example to explain the calculated expected results. The detailed statistical features are shown in Figure 2. There are thirteen countries in South America: Colombia, Venezuela, Guyana, Surinam, Ecuador, Peru, Brazil, Bolivia, Chile, Paraguay, Uruguay, Argentina, and French Guiana.

The number of routes, the number of airlines, and the number of aircraft types declined in 2019. Among them, the number of routes dropped the most obvious, directly from 118 in 2019 to 62 in 2020, a drop of 47.5%, before recovering to 77 in 2021. The longest route is from Caracas to Buenos Aires, 5,124 km; The shortest route is 187 km from Punta Arenas to Rio Gallegos. In terms of average flight distance, the average flight distance of all airlines was 2,032.69 km in 2019 but decreased to 1,932.3 km in 2020.

### Overall emissions of the six pollutions during 2019–2021

**Figure 3:**
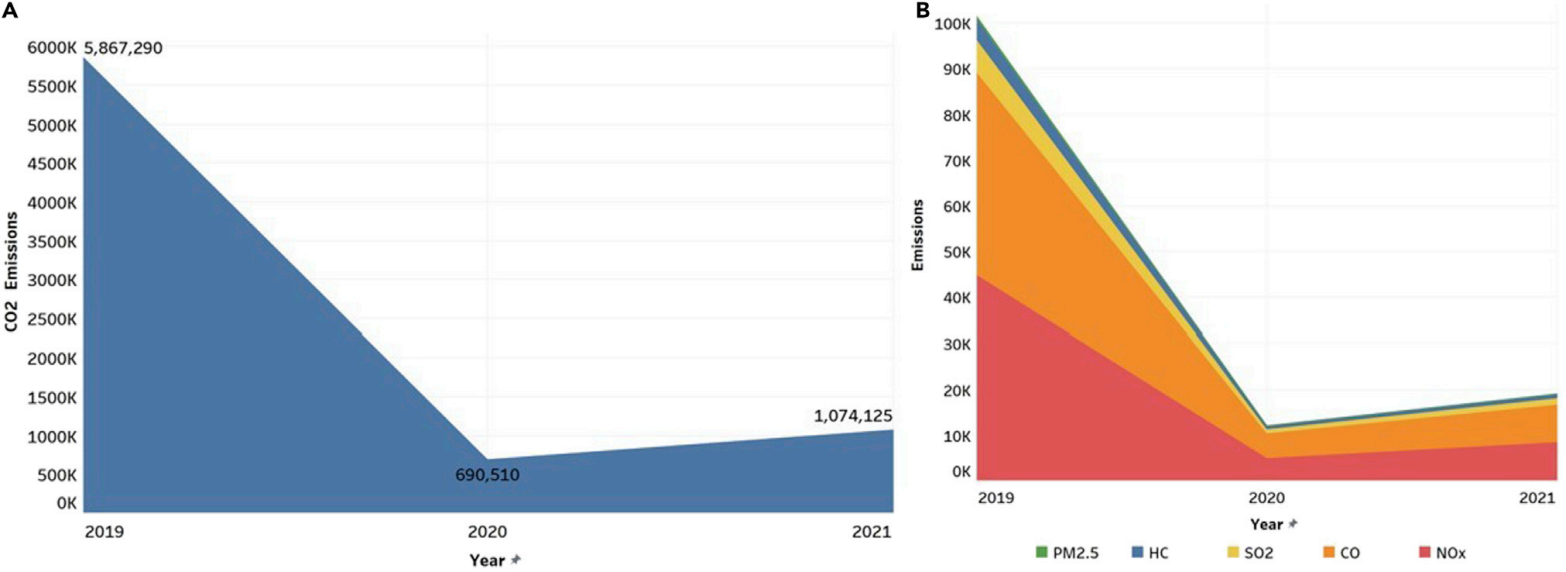
The overall emissions of the six pollutions during 2019–2021 (A) CO_2_ emissions. (B) Non-CO_2_ emissions.

According to Figure 3A, we can find that the carbon dioxide emissions from 2019 to 2021 are 5,867,289.72 tons, 690,510.49 tons, and 1,074,125.35 tons, respectively. Similarly, the emissions of the other five gases are shown in Figure 3B. Comparing Figures 3A and 3B, we can also find that carbon dioxide accounts for the most significant proportion of various pollutants. For example, in 2019, the overall carbon dioxide emissions were 5,867,289.72 tons, far exceeding the second gas, Nitrogen Oxide (44,985.09 tons). In addition to CO_2_, CO and NOx are also relatively large. However, the proportion of non-carbon dioxide pollution varies in different years. For example, in 2019 and 2021, the content of NOx is slightly higher than that of CO, but in 2020, the content of CO will exceed that of the former.

### Routes and airlines with the largest emissions of six pollutants during 2019–2021

**Figure 4:**
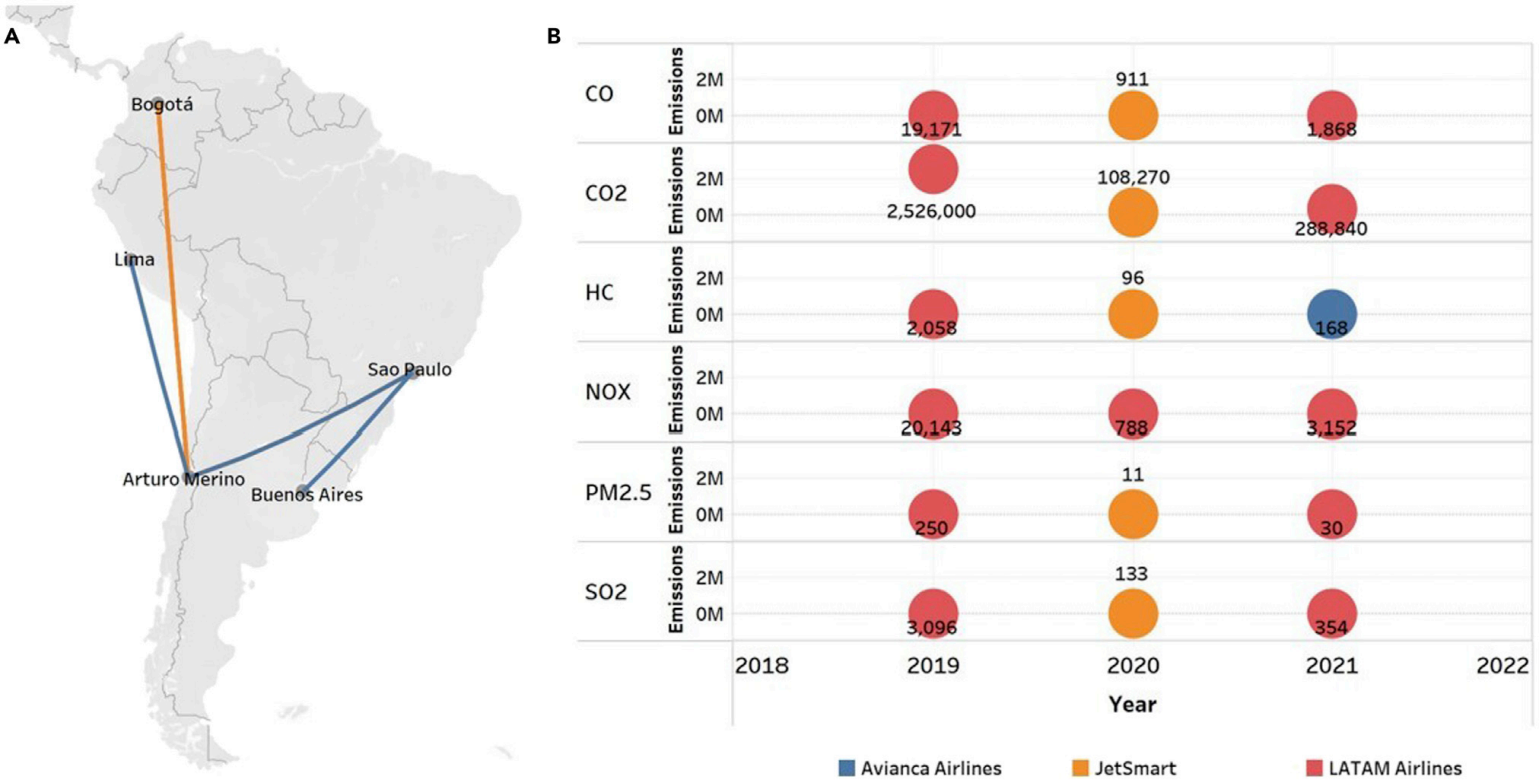
Routes and airlines with the highest emissions of six pollutants during 2019–2021 (A) The routes with the largest emissions. (B) The airlines with the largest emissions.

To further calculate the detailed emissions of six gases from international routes in South America in 2019–2021, we screened the routes and airlines with the most significant emissions of six gases in 2019–2021. The results are shown in Figure 4. Among them, the routes with the most critical emissions of CO_2_, CO, and SO_2_ in 2019 are Sao Paulo-Buenos Aires, the routes with the actual emissions of NO_X_ and PM2.5 are Lima-Arturo Merino, and the routes with the most significant emissions of HC are Sao Paulo-Arturo Merino; The routes with the most considerable HC emissions in 2020 are Sao Paulo- Arturo Merino, the other five routes with the most significant gas emissions are Bogot á-Arturo Merino; By 2021, the six routes with the most effective gas emissions are all Bogot á-Arturo Merino. It can be seen from Figure 4B that the airlines with the most critical annual emissions of CO_2_, CO, PM2.5, and SO_2_ from 2019 to 2021 are the same, that is, LATAM Airlines in 2019, JetSmart in 2020, and LATAM Airlines in 2021; The airlines that emitted the most NOX during the three years were all the same, LATAM Airlines; The airlines that emit the most HC differ in three years, that is, LATAM Airlines in 2019, JetSmart in 2020, and Avianca Airlines in 2021. In conclusion, LATAM Airlines has the highest frequency among the airlines with the most significant emissions, which is consistent with its status as one of the largest airlines in South America.

### Emission intensity in the CCD stage of six pollutants during 2019–2021

As mentioned earlier, we divide the distance into 11 segments based on the distance range: 0–500 km, 501 km-1,000 km, 1,001 km-1,500 km, 1,501 km-2,000 km, 2,001 km-2,500 km, 2,501 km-3,000 km, 3,001 km-3,500 km, 3,501 km-4,000 km, 4,001 km-4,500 km, 4,501 km-5,000 km, and 5,001 km-5,500 km. Furthermore, we considered the difference between sub-series, such as 320-214 and 320-232. Then, we get the aircraft’s emission intensity of the six pollutions from 2019 to 2021 based on the Modified BFFM2-FOA-FPM Method. Among them, the aircraft with the highest emission intensity in each distance segment and their intensities in 2019 are listed in Table 2. The emission intensity of other years can also be obtained through the supplemental tables. The proposed methods can analyze the emission intensity differences of different aircraft in the same series in detail.

**Table 2:** The aircraft with the highest emission intensity of six pollutants in each distance segment and its intensity in 2019

Year	Segment	CO_2_		CO		HC		NOx		PM2.5		SO_2_	
2019	0–500	320-232	8.E-02	320-232	7.E-04	320-232	8.E-05	320-232	6.E-04	320-232	9.E-06	320-232	1.E-04
	501–1000	320-233	3.E-02	320-233	2.E-04	320-233	2.E-05	320-233	2.E-04	320-233	3.E-06	320-233	3.E-05
	1001–1500	777-200ER	5.E-02	777-200ER	3.E-04	777-200ER	3.E-05	787-8	5.E-04	777-200ER	4.E-06	777-200ER	6.E-05
	1501–2000	777-300ER	4.E-02	777-300ER	3.E-04	777-300ER	3.E-05	777-300ER	5.E-04	787-8	3.E-06	777-300ER	5.E-05
	2001–2500	777-200ER	2.E-02	777-200ER	1.E-04	767-300ER	2.E-05	787-8	3.E-04	787-8	2.E-06	777-200ER	3.E-05
	2501–3000	777-300ER	3.E-02	777-300ER	3.E-04	777-300ER	3.E-05	787-8	4.E-04	787-8	3.E-06	777-300ER	4.E-05
	3001–3500	777-300ER	3.E-02	777-300ER	3.E-04	777-300ER	3.E-05	777-300ER	4.E-04	777-300ER	2.E-06	777-300ER	4.E-05
	3501–4000	767-300ER	2.E-02	767-300ER	1.E-04	767-300ER	2.E-05	767-300ER	2.E-04	767-300ER	2.E-06	767-300ER	2.E-05
	4001–4500	330-243E	2.E-02	767-300ER	1.E-04	767-300ER	2.E-05	787-9	3.E-04	787-9	2.E-06	330-243E	2.E-05
	4501–5000	319-113	1.E-02	319-113	9.E-05	319-113	1.E-05	319-113	7.E-05	737-800	1.E-06	319-113	1.E-05
	5001–5500	340-313E	4.E-02	340-313E	3.E-04	340-313E	4.E-05	340-313E	3.E-04	340-313E	4.E-06	340-313E	4.E-05

To show the data more clearly, we selected the aircraft with the highest emission intensity in each distance segment in 2019. As shown in Table 2, the aircraft with the highest emission intensity of six pollutants in the 0–500 segment in 2019 are 320-232. And emission intensity of CO_2_, CO, HC, NOx, PM2.5 and SO_2_ is 8. E-02 tons. km-1, 7.E-04 tons. km-1, 8.E-05 tons. km-1, 6.E-04 tons. km-1, 9.E-06 tons. km-1, and 1.E-04 tons. km-1 respectively.

### Accuracy analysis

In this section, we discuss the accuracy of the results. We have not found the direct data of international routes in South America, and we can only deduce it through indirect data. According to flight frequency, aircraft type, flight distance, aircraft weight, and other data, we calculated that the total turnover of international routes in South America in 2019 was 5,871,759,805.56 ton-km. According to the Civil Aviation Administration of China, the fuel consumption per ton-km is about 0.29–0.32 kg/ton-km.^27^ Multiplied by the emission coefficient of 3.157, the emission of South American international routes in 2019 was about 5,375,772.26 tons-5,931,886.63 tons. Our calculation result is 5,867,289.72 tons, the error rate is 1.09%–9.14%, and the average error is 5.115%. Considering the errors in the statistical process, the accuracy of the calculation method in this paper is relatively high.

## Limitations

It should be noted that the overall emissions are calculated through the standard LTO stage. Therefore, the emissions due to delays are not considered, and the aircraft transfer caused by temporary weather is not considered. Therefore, further investigation can calculate the emissions caused by delay, aircraft transfer, and air cargo.

## Troubleshooting

### Problem 1

How to determine the specific flight time?

Flight time is crucial to the calculation of emission intensity. Still, in the app of Variglight, on the same route, even for the same aircraft type, the specific flight times of different airlines may be quite other, and how determining flight time is the key to the accuracy of the calculation.

### Potential solution

Collect data for multiple weeks and take the median flight time as the specific flight time.

### Problem 2

Differences in weekly flight arrangements.

The airline’s arrangements may not be the same every week. Sometimes there are flights this week, but there are no flights next week, or the aircraft types and frequencies of flights are changed.

### Potential solution

Collect more data for a few weeks to see the overall situation. It is ignored if there are only one or two flights in three consecutive months. In terms of model and frequency, the model and frequency with the highest occurrence probability in three successive months are also taken as the calculation basis.

### Problem 3

Incomplete information on Variglight.

At present, the Variglight is a convenient channel to obtain the detailed information of aircraft routes. When collecting data on the Variglight, we would encounter a lack of aircraft, flight time, flight distance, etc. This rarely happens, but sometimes the relevant data is critical.

### Potential solution

If there is a lack of aircraft information, collect it for a few more weeks. If there is, follow the other weeks. If it is still lacking, replace it with the corresponding aircraft with the lowest carbon emission intensity according to the flight distance.

If there is a lack of flight time, collect more for a few weeks. If not, estimate according to the speed and flight distance of the aircraft.

If the flight distance is missing, enter the coordinates of the origin and destination airports on the map to calculate the 3-D distance.

### Problem 4

The Variglight may restrict access.

When collecting data on the Variglight, because of the significant frequency of access, IP access is often restricted, affecting the progress of data collection.

### Potential solution

There is no other way to deal with it. You can only pause the collection until the IP access restriction is lifted.

### Problem 5

Some flights may temporarily stop at the standby airport or temporarily add a transit airport.

When collecting the flight data, some flights may temporarily choose alternate airports or add transit airports due to weather or air traffic control reasons.

### Potential solution

Take it as a special case and ignore it, and use the information of normal flights as the information of this route.

## Resource availability

### Lead contact

Further information and requests should be directed to the lead author, Qiang Cui (cuiqiang@seu.edu.cn).

### Materials availability

This study did not generate new unique materials.

## Data Availability

The CCD and LTO emissions of each route and airline for the six pollutions are shown in Tables S1, S2, and S3. For example, the first data in Table S1 represents the LTO emissions and CCD emissions of six pollutants on the Cali-Caracas route of Avior Airlines. The overall emissions of the airline on the route are the sum of the LTO emissions and CCD emissions. The emission intensities of the six pollutions of each aircraft type can be found in Tables S4, S5, and S6. For example, the first data in Table S4 shows the CO2 emission intensity of each aircraft type in the 0–500 segment. The data can also be obtained in the Aviation Emissions Accounting Databases: http://www.aeads.com.cn. This paper does not use code. Any additional information required to reanalyze the data reported in this paper is available from the lead contact upon request.

## Supplemental information

Table S1. The emissions of six pollutions in 2019, related to step-by-step method details

Table S2. The emissions of six pollutions in 2020, related to step-by-step method details

Table S3. The emissions of six pollutions in 2021, related to step-by-step method details

Table S4. The emission intensities of the six pollutions in 2019, related to step-by-step method details

Table S5. The emission intensities of the six pollutions in 2020, related to step-by-step method details

Table S6. The emission intensities of the six pollutions in 2021, related to step-by-step method details
